# Description of the molecular and phenotypic spectrum in Chinese patients with aggrecan deficiency: Novel *ACAN* heterozygous variants in eight Chinese children and a review of the literature

**DOI:** 10.3389/fendo.2022.1015954

**Published:** 2022-10-28

**Authors:** Shuyun Deng, Lele Hou, Dan Xia, Xiaojuan Li, Xiaofang Peng, Xiaoqin Xiao, Jieming Zhang, Zhe Meng, Lina Zhang, Nengtai Ouyang, Liyang Liang

**Affiliations:** ^1^ Cellular & Molecular Diagnostics Center, Sun Yat-sen Memorial Hospital, Sun Yat-sen University, Guangzhou, China; ^2^ Department of Pediatrics, Sun Yat-sen Memorial Hospital, Sun Yat-sen University, Guangzhou, China

**Keywords:** short stature, *ACAN* mutation, genotype-phenotype correlation, phenotypic spectrum, growth-promoting treatment

## Abstract

**Objective:**

This study analyzed eight Chinese short stature children with aggrecan deficiency, and aimed to investigate potential genotype–phenotype correlations, differences in clinical characteristics between the Chinese and the Western populations, and effectiveness of recombinant human growth hormone therapy in patients with *ACAN* variants through a review of the literature.

**Methods:**

Pediatric short stature patients with *ACAN* heterozygous variants were identified using whole-exome sequencing. Subsequently, a literature review was carried out to summarize the clinical features, genetic findings, and efficacy of growth-promoting therapy in patients with *ACAN* variants.

**Results:**

We identified seven novel *ACAN* mutations and one recurrent variant. Patients in our center manifested with short stature (average height SDS: -3.30 ± 0.85) with slight dysmorphic characteristics. The prevalence of dysmorphic features in the Chinese populations is significantly lower than that in the Western populations. Meanwhile, only 24.24% of aggrecan-deficient Chinese children showed significantly advanced bone age (BA). Promising therapeutic benefits were seen in the patients who received growth-promoting treatment, with an increase in growth velocity from 4.52 ± 1.00 cm/year to 8.03 ± 1.16 cm/year.

**Conclusion:**

This study further expanded the variation spectrum of the *ACAN* gene and demonstrated that Chinese children with short stature who carried *ACAN* heterozygous variants exhibited early growth cessation, which may remain unnoticed by clinicians as most of these children had very mild dysmorphic characteristics and showed BA that was consistent with the chronological age. Genetic testing may help in the diagnosis.

## Introduction

*ACAN* gene encodes aggrecan, an important proteoglycan component of the cartilage extracellular matrix (1, 2). *ACAN* heterozygous variants can cause spondyloepiphyseal dysplasia, kimberley type (SEDK) (3); short stature and advanced bone age, with or without early-onset osteoarthritis (OA) and/or osteochondritis dissecans (OCD), while homozygous or compound heterozygous mutations in *ACAN* are associated with spondyloepimetaphyseal dysplasia, aggrecan type (SEMD) (4).

Recently, many genetic variants associated with short stature have been discovered through whole-exome sequencing (WES). Among these, pathogenic mutations of the *ACAN* gene play a substantial role in the failure of growth in the affected individuals (5–7). However, the correlation between genotype and phenotype is still unclear as the reported data were largely derived from small family pedigrees. Western populations, mainly of European descent, showed a majority of *ACAN* gene variants, whereas data retrieved from Chinese patients were limited. Although bone age (BA) characteristics varied substantially across populations (5), a systematic comparison of the differences in phenotypic features of *ACAN* variants between the Chinese and the Western populations has not been performed yet.

Here we report seven novel *ACAN* gene mutations and one recurrent variant in Chinese short stature children. This study focuses on the genotype-phenotype correlation and the differences in the phenotypic features between the Chinese and the Western patient cohorts with *ACAN* variants by conducting a comprehensive literature review. We also assess the effectiveness of recombinant human growth hormone (rhGH) treatment on patients with *ACAN* heterozygous variants.

## Materials and methods

### Patients’ information

Children with short stature of unknown etiology who visited the Pediatric Endocrinology Department in Sun Yat-sen Memorial Hospital of Sun Yat-sen University from January 2018 to April 2022 were enrolled based on the following inclusion criteria: (i.) abnormal limb proportions/skeletal dysplasia, (ii.) small for gestational age without catch-up growth, (iii.) multiple pituitary hormone deficiency with a specific phenotype, (iv.) isolated growth hormone deficiency with a family history of short stature or a specific phenotype, (v.) height below -3 SD of either the average height for corresponding age and sex or the parental genetic target height. Ultimately, 123 patients who met one or more of the enrollment principles underwent WES. *ACAN* heterozygous variants were detected in 8 out of the 123 patients tested. No pathogenic variants were observed in other genes associated with short stature in these eight patients. These patients’ clinical information were collected, including clinical manifestations, family history, biochemical and radiological materials, and treatment.

The study was performed with the approval of the Ethics Committee of Sun Yat-sen Memorial Hospital of Sun Yat-sen University (Ethical code: SYSEC-KY-KS-2022-060). Written consent with signature was taken from the probands and their parents for the above genetic research.

### Genetic analysis

Samples of peripheral blood were taken from the patients and affected family members. Genomic DNAs extraction and sequencing libraries preparation were performed with Illumina DNA Prep with exome v2 Enrichment kit (Illumina, USA) according to the manufacturer’s instructions. Illumina Nextseq550 (San Diego, CA, USA) was used for 150bp paired-end sequencing. Approximately 12GB of data per individual was obtained, and over 97.5% of the targeted regions (exonic regions of all nuclear genes along with +/-20bp of exon-intron boundaries) were sequenced more than 20 times, with an average coverage depth of ~150X.

After sequencing, the quality control assessment of raw reads was performed using the FastQC toolkit, followed by aligning the reads to the human reference genome hg19 with the Burrows-Wheeler Aligner (BWA). BAM processing was performed using the Picard tool and then realignment of indel regions, recalibration of base qualities, and variant detection and calling were performed using the Genome Analysis Toolkit (GATK) to produce variant call format (VCF) files. Finally, the VCF files were further annotated using ANNOVAR.

Variants meeting quality/coverage depth (≥10) and minor allele frequency (MAF<0.05%) were considered acceptable. This was followed by filtering the variants based on the patient’s phenotype, inheritance mode, clinical significance, and reported clinically relevant variants.

### Bioinformatic analyses of *ACAN* variants

Identified variants were checked against multiple pathogenic variants databases, such as Human Gene Mutation Database (HGMD, https://www.hgmd.cf.ac.uk), Leiden Open Variation Database (LOVD, https://databases.lovd.nl) and Clinvar Database (https://www.ncbi.nlm.nih.gov/clinvar). Pathogenicity prediction analysis of the identified *ACAN* variants was conducted by in silico prediction tools, such as SIFT (8), PolyPhen-2 (9), MutationTaster (10) and CADD V1.3 (http://cadd.gs.washington.edu/). At the same time, we used the the Genome Aggregation Database (gnomAD) to assess the population frequencies of these mutations. Rare variants were evaluated and classified following the 2015 American College of Medical Genetics and Genomics (ACMG) guidelines (11).

### Protein three-dimensional structure modeling

The protein structure database AlphaFold (https://www.alphafold.ebi.ac.uk/) was used to predict the aggrecan protein data bank (PDB) file. AlphaFold produces a per-residue confidence score between 0 and 100, providing precise estimates of the protein model’s reliability (12). A higher score denotes higher prediction accuracy of 3D protein structure. The confidence score of L (86^th^ amino acid) and T (288^th^ amino acid) of aggrecan is pretty high (94.85 and 94.49, respectively). Pymol (https://www.schrodinger.com/products/pymol) was used to visualize the 3D models of aggrecan with the p.Leu86Arg and p.Thr288Ile variants and perform point mutation analysis based on the above protein PDB file.

### Literature review and statistical analysis

Articles reporting *ACAN* variants associated with short-stature were searched on PubMed (up to April 30, 2022), and a literature review was performed on relevant clinical data in patients, particularly about the height standard deviation score (SDS), phenotypic characteristics, and genetic features.

The statistical analysis was completed with R and height was transformed into SDS for sex and chronological age (CA). Height SDS in different groups was compared using the t-test of two independent samples after identification of the data as normally distributed with equal variance. Differences in clinical characteristics between the Chinese and the Western populations were analyzed using Fisher’s exact test. Correlation analysis was conducted using Spearman’s rank correlation test. *P* value < 0.05 was considered statistically significant for all the tests.

## Results

### Clinical information

**Figure 1:**
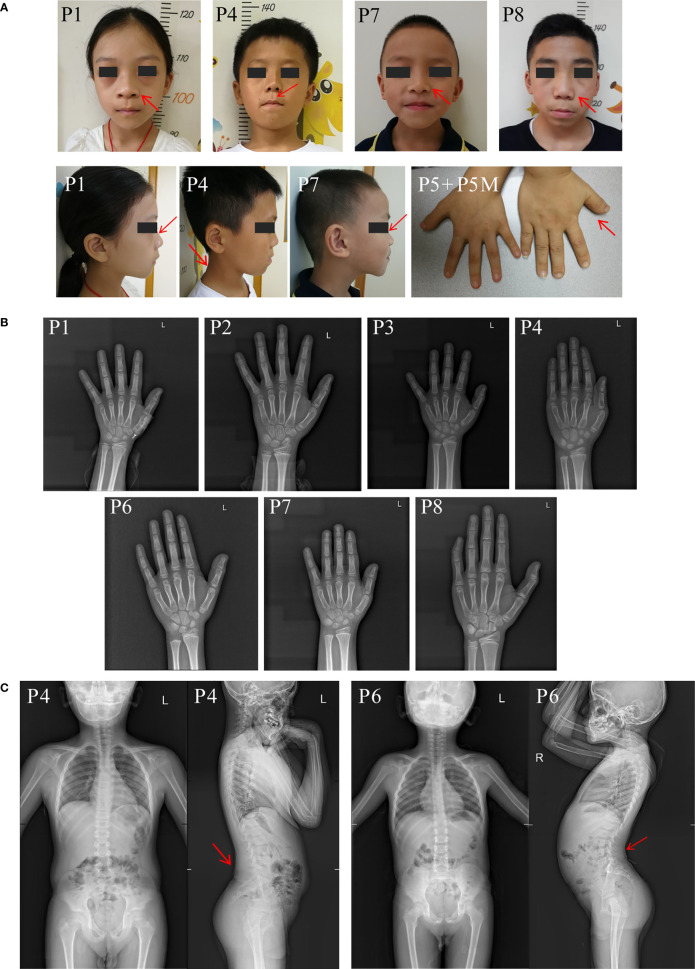
Clinical characteristics of the probands in our center. **(A)** Clinical pictures of our patients and their affected families. Red arrow symbols indicated the mild dysmorphic features of the patients. Patient numbers were indicated on the left upper corner of each plot. **(B)** Bone age radiographs of some affected patients’ left hands. **(C)** Spinal radiographs of probands patient 4 and patient 6. The patient’s identification is indicated in the top-left corner.

The eight unrelated probands (six males and two females) in the current study ranged in age from 5.9 to 14.5 years. The height SDS of probands ranged from -4.3 to -1.5 (average height SDS: -3.30 ± 0.85). At first visit, patient 8 was in Tanner stage G3/PH3 with a testicular volume of 12 ml, while others were in pre-pubertal stage. Phenotypic characteristics caused by *ACAN* heterozygous variants varied greatly among individuals (**Table 1**). Meanwhile, they had very mild dysmorphic features with somewhat hypertelorism (4/8), broad nose (4/8), long philtrum (2/8), flat nasal bridge (2/8), brachydactyly (1/8) and short neck (1/8) (**Figure 1A**). Furthermore, no arthritis or joint deformity was noticeable in these patients. BA was evaluated for seven probands according to Greulich and Pyle, TW3-RUS, and TW3-Carpal, respectively (**Figure 1B**, **Table 1**). Among them, the BA of two patients was advanced by 1.1 years (P6) and 1.9 years (P7), while the other five patients had BA that was consistent with CA. Additionally, patients in two families (P4, P6) had severe lumbar lordosis and/or scoliosis (**Figure 1C**).

**Table 1:** Clinical features of patients.

Patient	P1	P 2	P3	P4	P5	P6	P7	P8
*ACAN* mutation*	c.845C>T p.Thr282Ile	c.257T>G p.Leu86Arg	c.902G>A p.Trp301*	c.6176del p.Pro2059Leufs*2	c.7293del p.Cys2432Alafs*139	c.430G>C p.Ala144Pro	c.2023C>T p.Arg675*	c.2T>C p.?
Gender	Female	Male	Male	Male	Female	Male	Male	Male
Age at measurement (years)	7.5	7.5	6.3	6.6	8	7.2	5.9	14.5
Height (cm/SDS)	105.8/ -3.8	119.3/ -1.5	106/ -2.9	104.5/ -3.3	111/ -3.2	106.8/ -3.6	100.4/ -3.8	138.7/ -4.3
Weight (kg/SDS)	16.5/-2.8	23.6/ -0.6	19/-1.1	17.5/-1.7	NA	18.5/-2	15/-2.5	38/-1.9
Birth Length (cm/SDS)	50/ +0.18	NA	50/ -0.22	48/ -1.33	NA	48/ -1.33	48/ -1.33	50/ -0.22
Birth Weight (kg)	2.5	NA	3	2.7	NA	2.9	2.8	3
SGA	N	N	N	Y	NA	Y	Y	N
BA (Greulich-Pyle)	7.6	8.3	6.5	7.5	NA	8.3	7.5	13.5
RUS BA (TW3-RUS)	6	8.8	4.9	6.2	NA	7.3	6.6	13.7
Carpal BA (TW3-Carpal)	6.6	8.6	7	6.7	NA	8.3	7.5	12.9
GH peak (ng/ml)	23	12.5	5.53	10.20	NA	4.2	19.9	8.58
Head/Neck deformity	hypertelorism, broad nose, flat nasal bridge	-	-	hypertelorism (slight); broad nose; long philtrum; short neck	-	-	hypertelorism (slight); broad nose; flat nasal bridge	hypertelorism; broad nose; thin lips; long philtrum
Arthritis	-	-	-	-	-	-	-	-
Joint deformity	-	-	-	-	-	-	-	-
Spinal deformity	-	-	-	lumbar lordosis; scoliosis	-	lumbar lordosis	-	-
Hands/Feet deformity	-	-	-	-	short thumbs	-	-	-
Chest deformity	pectus excavatum	-	-	barrel-shaped chest	-	-	-	-

SDS, standard deviation score; NA, not available; N, no; Y, yes; SGA, small for gestational age; BA: bone age; CA: chronological age; *GRCh37, NM_013227.3.

### Genetic findings and functional assessment

**Figure 2:**
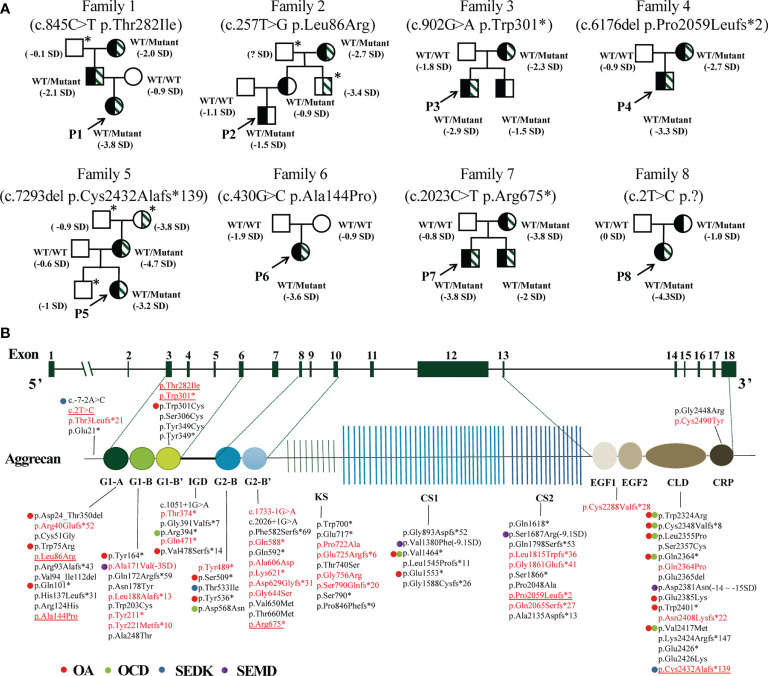
Pedigrees of the probands and variant spectrum of the *ACAN* gene. **(A)** Pedigrees of the eight families with heterozygous mutations in the *ACAN* gene. The height and height standard deviation scores of each individual are indicated. Half-black symbols indicate family members with *ACAN* heterozygous mutations. Half-shaded symbols indicate short stature (< -2SD). The arrows indicate the proband. The asterisks indicate that a blood sample was unavailable. The symbol “?” indicates an unknown phenotype. **(B)** Schematic of the aggrecan protein and the positions of variants in our study and reported before. All variant descriptions were checked applying the Mutalyzer program (https://mutalyzer.nl). *ACAN* structure based on RefSeq NM_013227.3 is shown in the top row with exon numbers marked in the green blocks. Aggrecan protein structure is shown in the bottom row with crucial domains. The variants detected in Chinese patients are highlighted in red, among which variants identified in our center are underlined. The graph does not indicate a balanced reciprocal translocation t(10;15)(q22.3;q26.1). Dots of different colors indicate diseases associated with the variants. G, globular domain; IGD, interglobular domain; KS, keratin sulfate attachment domain; CS, chondroitin sulfate attachment domain; EGF, epidermal growth factor-like domain; CLD, C-type lectin domain; CRP, complement regulatory like domain. OA, osteoarthritis; OCD, osteochondritis dissecans; SEDK, spondyloepiphyseal dysplasia, Kimberley type; SEMD, spondyloepimetaphyseal dysplasia, aggrecan type.

Eight different *ACAN* variants were identified in eight unrelated families (**Table 2**). Except for p.Arg675* in patient 7 (13), other variants have not been reported previously. There was one *de novo* mutation (p.Ala144Pro), and others were inherited from the affected parents (**Figure 2A**). 15 out of 19 individuals with *ACAN* heterozygous variants exhibited short stature (<-2SD), revealing that the pathogenic variants in the *ACAN* gene might display high penetrance. Furthermore, we noticed that the same variant could lead to significant differences in height within the pedigrees like families 2 and 8. These variants were widely scattered across the entire gene, including four variants located in the globular domain 1 (G1); while globular domain 2 (G2), C-type lectin-binding domain (CLD) of the globular domain 3 (G3), chondroitin sulfate attachment region (CS) and exon 2, the N-terminus of the protein each showed a single variant (**Figure 2B**).

**Table 2:** Genetic features of patients.

Patient	P1	P2	P3	P4	P5	P6	P7	P8
cDNA level	c.845C>T	c.257T>G	c.902G>A	c.6176del	c.7293del	c.430G>C	c.2023C>T	c.2T>C
Protein_level	p.Thr282 Ile	p.Leu86 Arg	p.Trp301*	p.Pro2059Leufs*2	p.Cys2432Alafs*139	p.Ala144 Pro	p.Arg675*	p.?
Inherited	Paternal	Maternal	Maternal	Maternal	Maternal	*De novo*	Maternal	Maternal
Variation type	Missense	Missense	Nonsense	Frameshift	Frameshift	Missense	Nonsense	start loss
Exon	6	3	6	12	16	3	10	2
Domain	G1-B’	G1-A	G1-B’	CS2	CLD	G1-A	G2-B’	-
SIFT	Del(0.001)	Del(0)	-	-	-	Tol(0.199)	-	-
Polyphen-2	Prob dam (1.0)	Prob dam (1.0)	-	-	-	Prob dam (0.999)	-	-
MutationTaster	Dis Caus (1.0)	Dis Caus (0.988)	-	-	-	Dis Caus (0.762)	-	-
CADD V3.1	24.9	24.1	-	-	-	21.2	-	-
Frequency in GnomAD	absent	absent	absent	absent	absent	absent	total:0.000003608	absent
ACMG classification	VUS	VUS	pathogenic	likely pathogenic	pathogenic	likely pathogenic	pathogenic	VUS

Del, deleterious; Tol, tolerated; Prob dam, probably damaging; Dis Caus, disease causing; CADD V1.3 values > 14 were classified as deleterious; VUS, variants of uncertain significance. The symbol (*) indicates translational termination.

**Figure 3:**
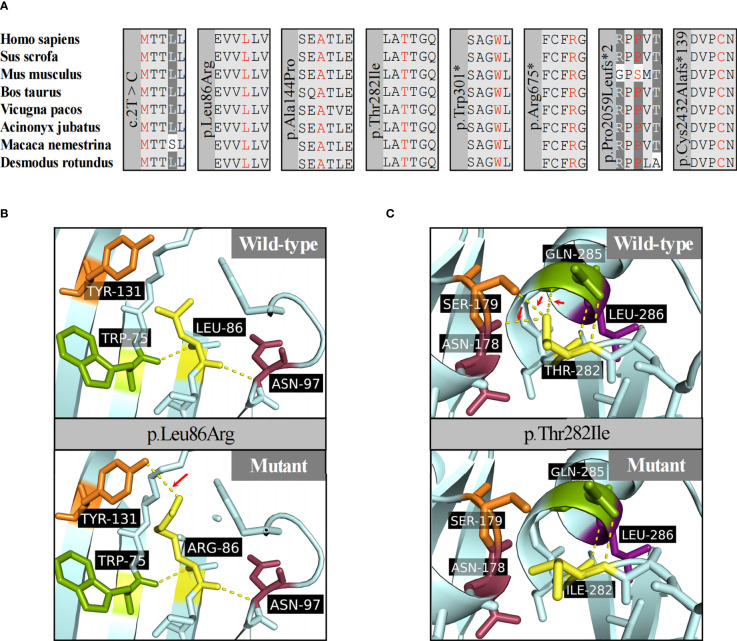
Protein function prediction of identified *ACAN* mutations in our center. **(A)** The amino acid highlighted in red represents the *ACAN* mutations in our patients among different species. **(B)** The three-dimensional models of wild-type and mutant aggrecan proteins for the variant p.Leu86Arg. **(C)** The three-dimensional models of wild-type and mutant aggrecan proteins for the variant p.Thr282Ile.

Truncating variants detected in this study were evaluated as likely pathogenic or pathogenic variants according to the ACMG guidelines. The mutation c.2T>C was considered as variant of unknown significance (VUS) due to the lack of functional *in vitro* studies, although it seemed to produce a more severe phenotype in P8 (height SDS = -4.3). To the best of our knowledge, this is the first report of a start loss variant in *ACAN*. The missense variants were predicted to be deleterious by at least three in silico tools, and the affected sites were highly conserved among different species (**Figure 3A**). The missense variants p.Ala144Pro, p.Thr282Ile, and p.Leu86Arg were located in the G1 domain, which is responsible for interaction with hyaluronan and link protein. The mutation p.Ala144Pro in P6 was classified as likely pathogenic (PS2 + PM2_supporting + PP3) based on the ACMG guidelines. For variant p.Leu86Arg in P2, a non-polar hydrophobic amino acid Leucine at position 86 was mutated to a hydrophilic (polar) Arginine, which was predicted to form an additional hydrogen bond with amino acid Tyrosine at position 131 near the C-terminal of the G1 domain (**Figure 3B**). Similarly, for variant p.Thr282Ile in P1, a basic polar neutral amino acid Threonine was replaced by non-polar hydrophobic Isoleucine at position 282, where its sidechain only formed two out of the five polar interactions observed in the wild type (**Figure 3C**). Thus, both were expected to destabilize the entire domain and affect its ligand binding properties. However, these two missense variants were not assessed further for functionality; therefore, they were considered VUS.

### Growth promoting treatment

Six out of eight probands received rhGH treatment, and the treatment duration was ranged from 5 months to 18 months. Patients 2 and 8 had received rhGH therapy previously. For P8, rhGH treatment was restarted at a dose of 49.5 μg/kg/day at the age of 14.7 years, but no advancement in height SDS was observed after five months of treatment. However, the other five individuals observed improved height SDS levels (from -3.28 ± 0.51 to -2.82 ± 0.34 SDS). Two patients (P1, P7) who received growth-promoting treatment >1 year showed some degree of improvement (average growth SDS > 0.5). The treatment materials are summarized in **Table 3**.

**Table 3:** Recombinant human growth hormone treatment of patients.

Patient	Gender	Protein change	Variation type	Age at GH introduction	Initial Height	GH duration (year)	Latest Height	Average growth SDS	GH dosage(μg/kg/day)
**P1**	Female	p.Thr282Ile	Missense	8.2	-3.8	1.1	-3.2	0.54	33-39.6
**P3**	Male	p.Trp301*	Nonsense	6.3	-2.9	0.7	-2.4	0.71	33
P4*	Male	p.Pro2059Leufs*2	Frameshift	6.6	-3.3	0.66	-3	0.45	56.1-62.7
				11.8	-2.5	0.3	-2.4	0.33	49.5
**P6**	Male	p.Ala144 Pro	Missense	8.2	-3.4	0.8	-2.9	0.62	36.3
**P7**	Male	p.Arg675*	Nonsense	6	-3.8	1.5	-3	0.53	56.1-62.7
**P8**	Male	p.?	Start loss	14.7	-4.1	0.4	-4.1	0	49.5

SDS, standard deviation score; GH, growth hormone. *Patient 4 discontinued the therapy after 8 months for some reasons, and then restarted at the age of 11.8 years. Average growth SDS = (Latest Height SDS - Initial Height SDS)/GH duration.

### Genotype-phenotype correlation

After a literature search, 190 affected individuals with *ACAN* variants had been reported among 125 families, of whom five patients had been diagnosed with SEMD (5, 6, 14–23). In total, 98 *ACAN* variants were identified, of which 34 variants were detected in Chinese patients, including those found in our center. All mutations were distributed across the entire *ACAN* gene without a hotspot region (**Figure 2B**), among which the mutation types included missense (35.71%), frameshift (28.57%), nonsense (26.53%), splicing (4.08%), in-frame deletion (3.06%), translocation (1.02%) and start loss (1.02%) mutations (**Supplementary Figure S1A**). Truncating variants, which lead to decreased protein production or loss of function, contributed to a significant portion of all *ACAN* variants. The majority of the variants located in the G1 domain (29.21%) and the G3 domain (21.35%), of which 17.98% occurred in the CLD domain of the G3 domain (**Figure 2B**, **Supplementary Figure S1B**).

Approximately 19.39% of the mutations were associated with OA/OCD, occurring mostly in G1 (n = 5) and CLD (n = 7) domains, while no OA/OCD was observed in KS and CS2 domains. Additionally, variants in the CLD domain were prone to cause OA and OCD simultaneously. Nevertheless, OA and OCD have been detected in individuals with missense mutations or truncating mutations. In other words, mutation type had little association with OA/OCD caused by *ACAN* variants.

**Figure 4:**
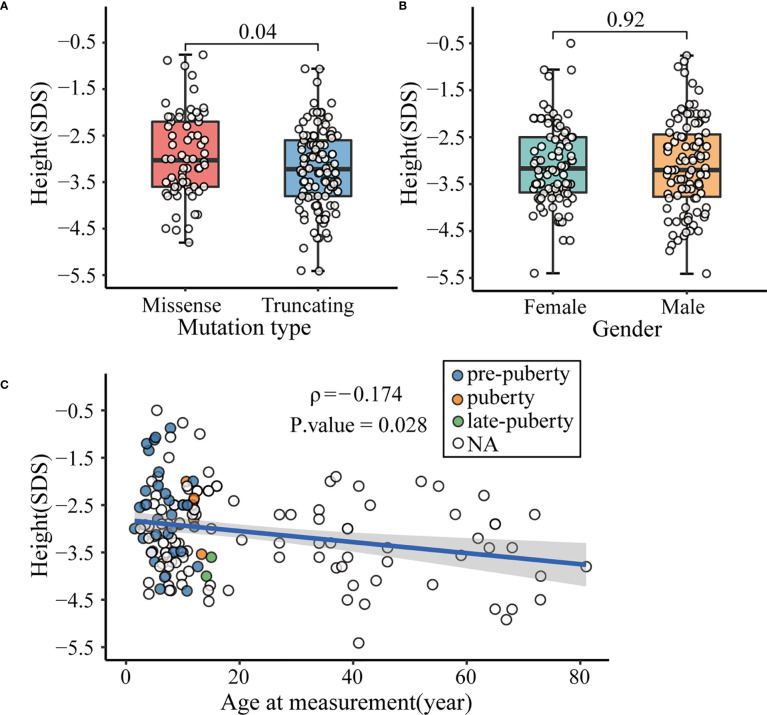
Factors affecting the height of patients with *ACAN* heterozygous mutations. **(A)** Comparison of height standard deviation score (SDS) in different mutation types. **(B)** Comparison of height SDS in different genders. **(C)** Correlation between height SDS and age at measurement. Dots with different color represent different pubertal status of the patients. White dot indicated that data of the patient’s pubertal status was not available, or the patient was already an adult. Subjects diagnosed with spondyloepimetaphyseal dysplasia, aggrecan type (SEMD) were excluded from the above analyses to avoid interference.

Concurrently, we summarized the detailed clinical characteristics of the affected subjects. Individuals with *ACAN* truncating variants were found significantly shorter than those with missense variants (-3.23 ± 0.87 SDS vs -2.94 ± 0.92 SDS, *P* = 0.04; **Figure 4A**), whereas no significant differences in height were seen between males and females (-3.10 ± 0.97 SDS vs -3.08 ± 0.86 SDS, *P* = 0.92; **Figure 4B**). Interestingly, a significant negative correlation was observed between age and height SDS (Spearman’s rank correlation coefficient ρ = -0.174, *P* = 0.028; **Figure 4C**).

### Phenotypic differences between the Chinese and the Western patients

As shown in **Table 4**, all patients with *ACAN* heterozygous mutations previously reported in the Western populations, including American, Caucasian, Dutch, Portuguese, and Spanish, were reviewed and compared with the clinical features of Chinese patients to access the phenotypic similarities and differences between the Chinese population and those of other ethnicities.

**Table 4:** Differences in clinical features between the Chinese and the Western patients.

Phenotype	Clinical Manifestations	Frequency in Chinese patients (3, 5, 18, 24–28)	Frequency inWestern patients (3, 6, 7, 13–15, 17, 19, 21, 22, 29–31)	P.value
Head/Neck deformity	Relative Macrocephaly	1/23 (4.35%)	6/24 (25%)	0.097
	Prominent Forehead	2/27 (7.41%)	17/54 (31.5%)	0.024
	Hypertelorism	4/20 (20%)	2/25 (8%)	0.383
	Mild Posteriorly Rotated Ears	0/20 (0%)	3/6 (50%)	0.008
	Flat Nasal Bridge	7/29 (24.1%)	13/34 (38.2%)	0.284
	Broad Nose and Philtrum	4/21 (19%)	4/24 (16.7%)	1
	Thin Lips	1/20 (5%)	4/24 (16.7%)	0.356
	High Palate	0/21 (0%)	6/27 (22.2%)	0.029
	Short Neck	5/28 (17.9%)	7/11 (63.6%)	0.017
	Midface Hypoplasia	5/27 (18.5%)	16/35 (45.7%)	0.032
Arthritis	Osteochondritis Dissecans	0/30 (0%)	14/62 (22.6%)	0.004
	Osteoarthritis	1/30 (3.33%)	23/52 (44.2%)	0.00004
Joint deformity	Elbow Deformity	0/20 (0%)	1/6 (16.7%)	0.231
	Feet Deformity (varus/valgus)	0/20 (0%)	2/6 (33.3%)	0.046
	Coxa Valga	0/21 (0%)	4/25 (16%)	0.114
	Patellar Luxation	0/20 (0%)	2/6 (33.3%)	0.046
Chest deformity	Chest Deformity	8/31 (25.8%)	7/U	NA
Spinal deformity	Lumbar Lordosis	5/28 (17.9%)	2/6 (33.3%)	0.580
	Hyperlordosis	0/21 (0%)	4/28 (14.3%)	0.125
	Scoliosis	4/26 (15.4%)	1/5 (20%)	1
	Disc Disease	0/20 (0%)	15/27 (55.6%)	0.00002
Hands/Feet deformity	Brachydactyly	4/27 (14.8%)	36/46 (78.3%)	0.0000001
	Short Metacarpals	1/26 (3.85%)	4/18 (22.2%)	0.142
	Short Thumbs	1/22 (4.55%)	3/6 (50%)	0.022
	Broad Thumbs	1/20 (5%)	7/21 (33.3%)	0.045
	Broad Great Toes	0/20 (0%)	6/11 (54.5%)	0.0006
Bone age	Markedly advanced	8/33 (24.24%)	23/55 (41.82%)	0.111
	Slight advanced	8/33 (24.24%)	6/55 (10.91%)	0.133
	Normal	10/33 (30.3%)	17/55 (30.91%)	1
	Slight delayed	1/33 (3.03%)	2/55 (3.64%)	1
	Markedly delayed	6/33 (18.18%)	7/55 (12.73%)	0.543

The ratios recorded for bone age are given as the number of individuals, while the other characteristics are the number of families. Chest deformity include barrel-shaped chest, pectus carinatum, pectus excavatum and rib valgus. U, denominator unknown; NA, data not available. Markedly advanced: BA-CA > 1; Slight advanced: 0.5 < BA-CA ≤ 1; Normal: - 0.5 ≤ BA-CA ≤ 0.5; Slight delayed: -1 ≤ BA-CA < -0.5; Markedly delayed: BA-CA < -1.

Primarily, the clinical characteristics of the patients with aggrecan deficiency vary significantly across the populations. Specifically, brachydactyly (78.3%), short neck (63.6%), disc disease (55.6%), broad great toes (54.5%), midface hypoplasia (45.7%), and OA (44.2%) were identified as the most represented clinical features in the Western population. In contrast, the incidence of these dysmorphic features was significantly lower in Chinese short stature patients. Furthermore, features like OCD, joint deformity, hyperlordosis, and disc disease were absent in Chinese patients.

Lastly, it is worth mentioning that 41.82% of the Western population showed an advanced BA >12 months, whereas only about 24.24% of the Chinese patients showed remarkably advanced BA. Moreover, more than half (57.58%) of the Chinese pediatric patients showed a BA that was consistent with CA (-1 ≤ BA-CA ≤ 1), which can also be observed in the patients in our center.

## Discussion

Recently, with the vigorous advancement of high-throughput sequencing technology, the application of WES in genetic diagnosis has led to the detection of many short stature patients with pathogenic variants in the *ACAN* gene. Here we identified eight heterozygous variants in *ACAN* with high penetrance in the affected families, all of which were novel mutations except for one reported previously, further expanding the *ACAN* mutation spectrum.

First, the literature review for all previously reported variants of *ACAN* suggests that the genotype-phenotype correlations are complex in individuals with aggrecan deficiency. Aggrecan is a large chondroitin sulphated proteoglycan that plays a crucial role in the growth plate cartilage (32, 33). The fixed negative charge of this proteoglycan attracts ions and water molecules, allowing the articular cartilage to resist compressive load with minimal deformation (34, 35). It is well known that the severity of short stature is related to the degree of growth plate involvement. Based on the comprehensive literature review, we demonstrate for the first time that the effect of truncating variants on height is more severe than that of missense variants, and the degree of severity may be gender-independent (**Figure 4**). Thus, the impairment of growth plate chondrogenesis is likely due to functional haploinsufficiency of aggrecan caused by the mutation types rather than the hormonal conditions. Additionally, most patients with heterozygous mutations in the CLD domain presented both OA and OCD (6, 15, 17, 29). The G3 domain interacts with tenascins and fibulins through CLD (34). Therefore, pathogenic variants in CLD may disrupt interactions between aggrecan and other matrix molecules in growth plate cartilage, resulting in a more severe phenotype affecting the growth plate and articular cartilage function. Furthermore, a gradual decline in height SDS with advancing age was observed. This might be due to the lack of growth spurt in patients with aggrecan deficiency; patients in late adolescent perhaps had a slower growth rate than those in pre-pubertal stage (**Figure 4C**). Besides, severe OA and intervertebral disc degenerative disease showed early onset in the affected families (6); this perhaps also accounted for some fraction of the observed association.

Next, the differences in clinical characteristics between the Chinese and the Western populations with *ACAN* heterozygous mutations are described for the first time. Brachydactyly, short neck, mild midface hypoplasia, disc disease, and OA were most often observed in patients with aggrecan deficiency (14, 36); however, these common phenotypic features in the Western population have a low prevalence in the Chinese population (**Table 4**). As seen in the cohort under study, P2 and P3 had no dysmorphic characteristics other than mild to moderate short stature; P5 and P6 only exhibited short thumbs and lumbar lordosis respectively (**Table 1**). It is consistent with previous descriptions of most Chinese individuals with heterozygous variants in the *ACAN* gene, who only manifested short stature without any other obvious abnormalities (5, 24–26). The deformity characteristics are not obvious in the Chinese populations, perhaps because Chinese people have flattened faces, and the special facial features of patients with aggrecan deficiency are not very distinguishable from those of normal. Abnormal facial characteristics may remain unnoticed by clinicians and not be described as deformity phenotypes. Although OCD, hyperlordosis and disc disease have been identified in the Western population, such skeletal disorders have not previously been reported in Chinese patients. This discrepancy may be ascribed to the intrinsic ethnic difference, and it may also due to the time node of OCD and disc disease has not been arrived in most of Chinese pediatric short stature patients reported previously. Besides, advanced BA has previously been considered a major characteristic in *ACAN* heterozygous variants carriers (7). Subsequently, normal or delayed BA was also identified in patients with *ACAN* heterozygous variants (5, 14, 17, 19, 22). According to our statistical results, most Western populations presented with an advanced BA >1 year. Consistently, about 64.52% (20/31) of aggrecan-deficient children showed markedly advanced BA (>1 year) in a large international cohort study (6). Conversely, a large portion of Chinese children with aggrecan deficiency did not show significantly advanced BA. Only 28.57% (2/7) of patients in this study had an apparent advanced BA. Specifically, we noted that BA measured by the TW3-RUS method was slightly delayed, whereas BA measured by the TW3-Carpal method tended to be older than CA (**Table 1**). Therefore, whether such differences in BA across populations can be attributed to intrinsic ethnic differences or differences in BA assessments among observers remains to be investigated.

Last, rhGH treatment is effective in patients with short stature caused by *ACAN* heterozygous variants, according to observations in our cohort study and previous reports (5, 24, 27, 37). We retrospectively analyzed the efficacy of rhGH in 29 short stature children with *ACAN* heterozygous variants (6, 14, 19, 24, 27, 29, 37, 38). The average treatment duration of these patients was 1.67 years (range 0.5-7.7 years), and the average height SDS before and after treatment was -2.48 ± 0.91 SDS and -1.81 ± 0.99 SDS, respectively. In our study, the average height SDS before treatment was -3.55 SDS (range -4.1 to -2.9 SDS), and it increased to -3.10 SDS (range -4.1 to -2.4 SDS) after a varying duration of treatment (**Supplementary Figure S2A**). Consistent with previous reports (5, 17), patients can achieve better therapeutic benefits during the first year of the treatment. The change of growth velocity (GV) within one year in our study was comparable to the therapeutic effect (GV range 5.21 ± 1.10 to 8.87 ± 1.53 cm/year) of a prospective trial of rhGH treatment in patients with *ACAN* heterozygous variants reported by *Muthuvel et al*. (37) (**Supplementary Figure S2B**). For the long-term effects of treatment, P1 and P7 who were treated for more than 1 year presented with some degree of improvement in height SDS; however, considering that patients have not achieved adult height, they need continued assessment and follow-up to evaluate the impact of a long duration of rhGH therapy on final height. Additionally, P8 who received treatment during puberty exhibited poor response; it is difficult to judge the efficacy of rhGH because P8 was treated during adolescence and the treatment duration was short.

The main limitation of the current study is that it was a retrospective single-center study with only 8 Chinese patients who carried the *ACAN* heterozygous variants. We thus further collected genetic and phenotypic information of 25 Chinese patients previously reported, which expanded the study population to some extent. However, due to the relatively small number of patients included, future analyses with larger case numbers are needed to confirm these findings.

In conclusion, this is the first report to reveal the differences in clinical characteristics between the Chinese and the Western populations with aggrecan deficiency. Our result support that Chinese pediatric short stature patients show no apparent dysmorphic features or skeletal abnormities, and most exhibited BA that were consistent with CA. This suggests that pediatric endocrinologists may overlook individuals with *ACAN* pathogenic variants; therefore, the prevalence of *ACAN* pathogenic variants in the Chinese population is likely to be underestimated. Diagnosis of this condition is difficult, and *ACAN* sequencing is helpful.

## Data availability statement

The original contributions presented in the study are included in the article/**Supplementary Material**. Further inquiries can be directed to the corresponding authors.

## Ethics statement

The studies involving human participants were reviewed and approved by the Ethics Committee of Sun Yat-sen Memorial Hospital of Sun Yat-sen University (Ethical code: SYSEC-KY-KS-2022-060). Written informed consent to participate in this study was provided by the participants’ legal guardian/next of kin. Written informed consent was obtained from the minor(s)’ legal guardian/next of kin for the publication of any potentially identifiable images or data included in this article.

## Author contributions

LL and SD conceived and organized this study and cowrote the first draft of the manuscript. LH and DX assisted organization and wrote the draft of the manuscript together. All authors listed were involved in patient consultation, data collection, and analysis. All authors read and approved the submitted version.

## Acknowledgments

We are grateful to the patients and their families for the participation in this study.

## Conflict of interest

The authors declare that the research was conducted in the absence of any commercial or financial relationships that could be construed as a potential conflict of interest.

## Publisher’s note

All claims expressed in this article are solely those of the authors and do not necessarily represent those of their affiliated organizations, or those of the publisher, the editors and the reviewers. Any product that may be evaluated in this article, or claim that may be made by its manufacturer, is not guaranteed or endorsed by the publisher.
