# Supplementary material for: Description of the molecular and phenotypic spectrum in Chinese patients with aggrecan deficiency: Novel ACAN heterozygous variants in eight Chinese children and a review of the literature
Source: Front Endocrinol (Lausanne). 2022 Oct 28;13:1015954. doi: 10.3389/fendo.2022.1015954 (PMC9649928; doi:10.3389/fendo.2022.1015954)
Supplement: Supplementary file 1 [file DataSheet_1.docx]

**Supplementary Materials**

**Supplemental Figure Legends**

**Figure S1. Proportions of different mutation types and domains among patients with heterozygous *ACAN* mutations.**

**(A)** Proportion of mutation types. **(B)** Proportion of mutation distribution in domains.

**Figure S2. Treatment efficacy in patients with *ACAN* heterozygous variants.**

**(A)** Mean growth velocity (± SD) in patients from two cohorts. Our data is represented by red line. Green line represents data as reported by *Muthuvel et al*. **(B)** Change in height SDS of patients (n=6) in our study after a varying duration of recombinant human growth hormone treatment.

**Supplemental Figures**


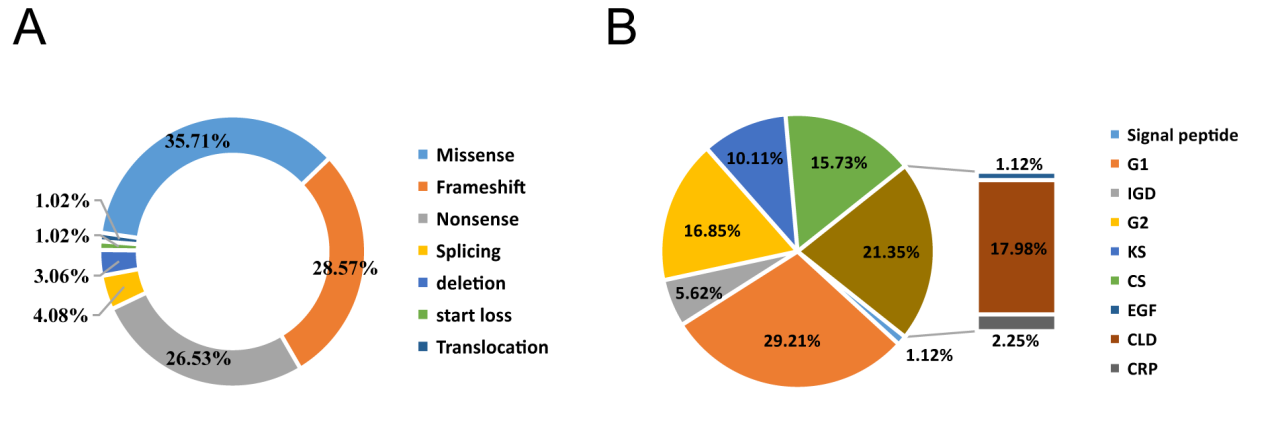


Figure S1. Proportions of different mutation types and domains among patients with heterozygous *ACAN* mutations.


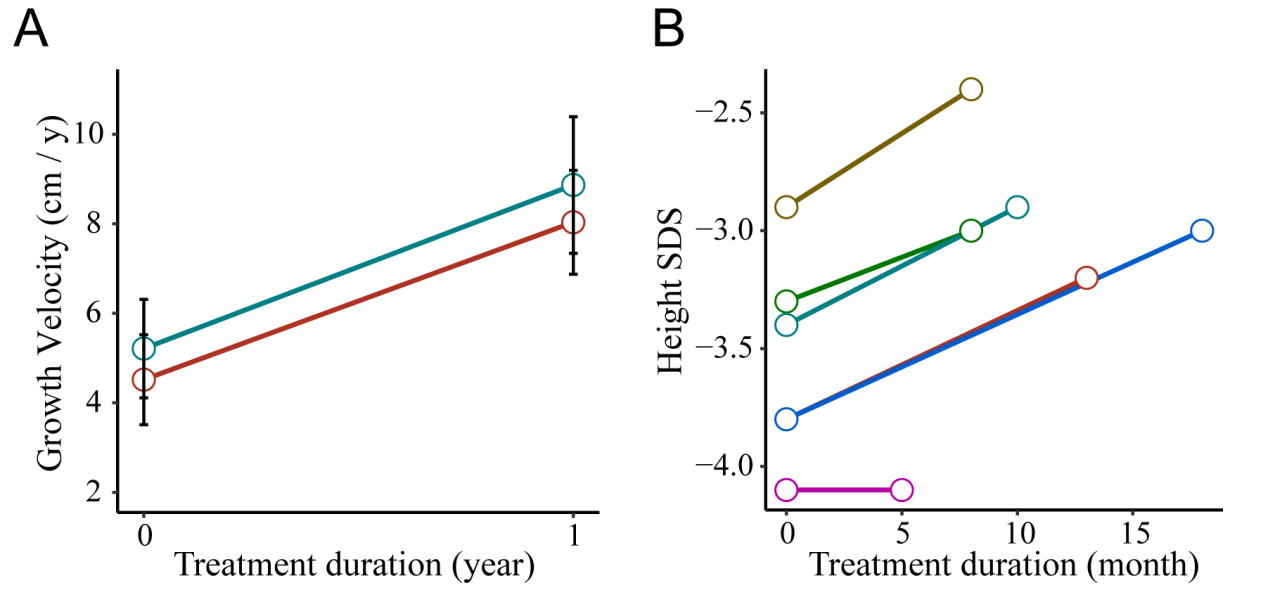


Figure S2. Treatment efficacy in patients with *ACAN* heterozygous variants.
